# Neural signals encoding shifts in beliefs

**DOI:** 10.1016/j.neuroimage.2015.10.067

**Published:** 2016-01-15

**Authors:** Philipp Schwartenbeck, Thomas H.B. FitzGerald, Ray Dolan

**Affiliations:** a The Wellcome Trust Centre for Neuroimaging, UCL, 12 Queen Square, London WC1N 3BG, UK; b Centre for Cognitive Neuroscience, University of Salzburg, Salzburg 5020, Austria; c Neuroscience Institute, Christian-Doppler-Klinik, Paracelsus Medical University Salzburg, Salzburg 5020, Austria; d Max Planck University College London Centre for Computational Psychiatry and Ageing Research, London WC1B 5EH, UK

**Keywords:** Dopamine, Information theory, Epistemic value, Belief formation, Psychosis

## Abstract

Dopamine is implicated in a diverse range of cognitive functions including cognitive flexibility, task switching, signalling novel or unexpected stimuli as well as advance information. There is also longstanding line of thought that links dopamine with belief formation and, crucially, aberrant belief formation in psychosis. Integrating these strands of evidence would suggest that dopamine plays a central role in belief updating and more specifically in encoding of meaningful information content in observations. The precise nature of this relationship has remained unclear. To directly address this question we developed a paradigm that allowed us to decompose two distinct types of information content, information-theoretic surprise that reflects the unexpectedness of an observation, and epistemic value that induces shifts in beliefs or, more formally, Bayesian surprise. Using functional magnetic-resonance imaging in humans we show that dopamine-rich midbrain regions encode shifts in beliefs whereas surprise is encoded in prefrontal regions, including the pre-supplementary motor area and dorsal cingulate cortex. By linking putative dopaminergic activity to belief updating these data provide a link to false belief formation that characterises hyperdopaminergic states associated with idiopathic and drug induced psychosis.

## Highlights

• Recent work suggests a central role of dopamine in (aberrant) belief formation.
• Using fMRI we show that dopamine-rich midbrain regions encode shifts in beliefs.
• Performance correlated negatively with the effect size of midbrain activation.
• Shifts in beliefs are dissociated from surprise signalled in prefrontal regions.
• These data provide a link to false belief formation in psychosis.

## Introduction

Adaptive behaviour mandates agents build models of their environment and use these to appropriately update their beliefs about the causes of sensory information, a process formalised within Bayesian brain theories (Dolan and Dayan, 2013, Friston et al., 2014b, Friston, 2010, Körding and Wolpert, 2004, Tenenbaum et al., 2006). It has long been proposed that dopamine plays an important role in belief formation and, crucially, in aberrant belief formation in psychosis (Corlett et al., 2009, Howes and Kapur, 2009, Howes et al., 2011a, Howes et al., 2011b, Roiser et al., 2013, Shaner, 1999). This view is supported by studies indicating that dopamine is critical for cognitive flexibility (Bestmann et al., 2014, Van Holstein et al., 2011) and task switching (Cools et al., 2009, Stelzel et al., 2010). It also resonates with observations showing dopamine neurons respond to both novel and unexpected stimuli (Bromberg-Martin et al., 2010, Bunzeck and Düzel, 2006, Horvitz, 2000, Iglesias et al., 2013) and reflect preferences for advance information (Bromberg-Martin and Hikosaka, 2009). These findings suggest that dopamine plays a central role in belief updating and, therefore, in encoding the meaningful information content of observations. Despite these lines of suggestive evidence, a relationship between dopamine signalling and belief-updating in humans has not been demonstrated so far.

To examine this question we defined two related, but distinct, measures of information content that could be signalled by dopamine. The first quantity is surprise or surprisal (Barto et al., 2013) and reflects the information content of a stimulus in a purely information-theoretic sense. For an observation *o* given a model *m* with parameters *θ* this is defined as:

$$-lnP\left(o|\theta ,m\right).$$

This is the negative log probability of an observation, which reflects the simple unexpectedness of an observation given an agent's model of the world. Importantly, this does not necessarily index the ‘usefulness’ of information to an agent, leading to the so-called ‘white noise’ paradox, where maximally informative stimuli are those that are entirely random and therefore meaningless for updating an agent's model about the world (Barto et al., 2013). Consequently, a surprising observation is not necessarily associated with improving an agent's beliefs about the environment.

This motivates an alternative definition of information in terms of actual shifts in beliefs. Formally, this corresponds to Bayesian surprise or the Kullback–Leibler divergence between prior and posterior beliefs about the world:

$$\left(KL\left[P\left(\theta |o,m\right)||P\left(\theta |m\right)\right]\right).$$

Bayesian surprise can be understood as meaningful information or the ‘epistemic value’ of an observation (Friston et al., 2015). This quantity has previously been used successfully to model visual search behaviour (Itti and Baldi, 2009) and exploration (Sun et al., 2011).

**Fig. 1:**
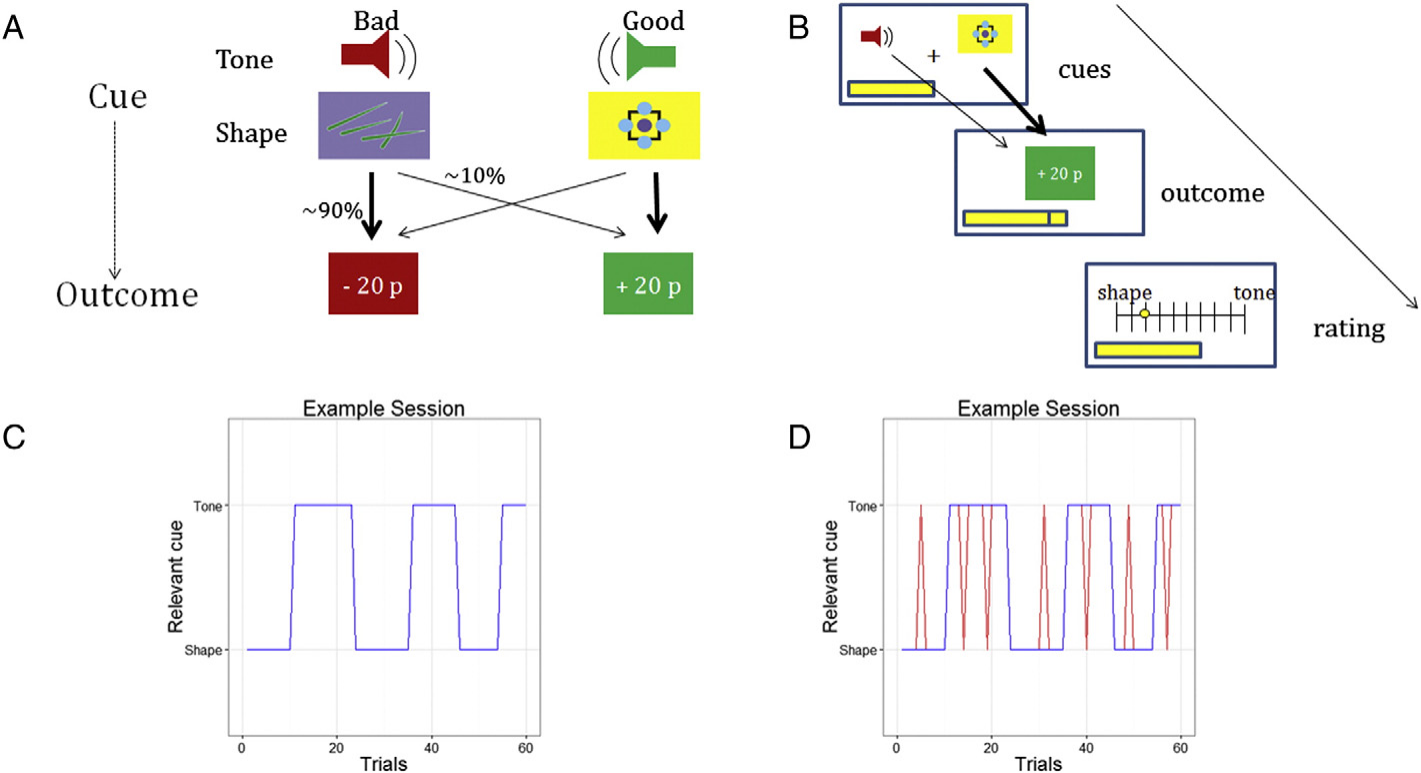
Task. (A) Subjects were instructed that throughout the experiment one ‘good’ shape and tone predicted a monetary win and one ‘bad’ shape and tone predicted a loss with 90% cue-validity. Monetary outcomes varied between ∓ 10 and ∓ 30 pence. (B) Each trial started with a simultaneous presentation of one shape (good or bad) and one tone (good or bad), followed by a trial outcome (win or loss). Based on this observation, subjects were asked to infer whether visual or auditory cues currently predict trial outcomes and indicate their belief on a rating bar. At any given trial, only one modality predicted the outcome. (C) Subjects were instructed that cue contingencies did not change on a trial-by-trial basis but periodically, with about 5–6 reversal of contingencies during one session (consisting of 60 trials). (D) Because cues had only 90% validity, sometimes unexpected trial-outcomes were observed (red line) without reflecting an actual shift in cue contingencies (blue line).

Since model updating occurs in response to unexpected observations, in many situations information-theoretic surprise and shifts in beliefs are highly correlated. However, they are decorrelated in situations where surprising stimuli do not lead to significant model updating, as occurs when an observation is similarly unlikely under all possible hypotheses. We exploited this fact to develop a task in which subjects were asked to infer, on a trial-by-trial basis, whether a visual or auditory cue determined the current probabilities of observing different trial outcomes (see Fig. 1a). Subjects were instructed, truthfully, that on any given trial outcomes were exclusively determined by one of the cue types (i.e., sensory modalities), and that this contingency would switch periodically. At the end of each trial, they were asked to rate their belief about the current contingency, on a visual analogue scale ranging from 1 (complete certainty that visual cues determined outcomes) to 11 (complete certainty that auditory outcomes did) (see Fig. 1b for an example trial). To allow us to clearly separate BOLD responses engendered by reward processing from those relating to information content, we used monetary gains and losses as outcomes. These outcomes determined subject's earnings at the end of the experiment, but were independent of performance on the rating task.

## Materials and methods

### Participants

27 subjects (16 females) with no known history of psychiatric or neurological disorders provided written informed consent and participated in the study. Participants had a mean age of 24.9 (standard deviation = 5.28, range = [19,43]) and were recruited from the University College London Psychology Subject Pool. Subjects were instructed that they would be paid between £25 and £35, determined by the sum of their earnings in one behavioural training session and three sessions in the scanner. The average payment of subjects was £33.78. The study was approved by the UCL Research Ethics Committee.

### Procedure

Participants underwent a 1-h training period followed by one and a half hours of scanning. In the experiment, subjects had to infer on a trial-by-trial basis whether auditory or visual cues currently determined probabilities of seeing a specific trial outcome, where the cue contingencies switched periodically (about 5 to 6 times in one session). Trial outcomes were wins or losses varying randomly between 10 and 30 pence.

Subjects were instructed that on any given trial only one of the two cue types (visual or auditory) predicted outcomes while the other had no predictive validity. In an initial training session subjects learned that in both the visual and auditory domains there were one “good” cue that was predictive of a monetary win and one ‘bad’ cue that was predictive of a monetary loss, where cues had a validity of 90%. Subjects were instructed that these contingencies would remain constant throughout the experiment, such that the identity of the ‘bad’ tone and shape, predictive of a monetary loss, and the ‘good’ tone and shape, predictive of a monetary win, remained the same. Crucially, therefore, participants were asked to perform inference on cue contingencies, not on the valence of cues (good or bad).

In the actual task, each trial started with a simultaneous presentation of one visual (good or bad) and one auditory (good or bad) cue, followed by a trial outcome (monetary win or loss). Based on this observation, subjects had to infer whether auditory or visual information was currently predictive of trial outcomes and rate their belief on a scale ranging from 1 (complete certainty that one modality determined outcomes) to 11 (complete certainty that the other modality did), where the sides on which ‘Shape’ and ‘Tone’ would appear on the rating bar were counterbalanced between subjects. Importantly, half of the trials were informative (one cue being predictive of a win whereas the other being predictive of a loss) whereas the other half was uninformative (both cues being good or bad). This introduced a crucial dissociation between unexpected events (i.e., information-theoretic surprise) and actual shifts in beliefs (Bayesian surprise) that could take place in informative trials only, while decorrelating these two measures of information content from actual reward.

To thoroughly train subjects on the contingencies of the task, one full training session was performed prior to the actual experiment in the scanner, preceded by a training session on the valence of the cues (i.e., learning which cue was predictive of a win/loss) and a session in which cues had 100% validity to familiarise subjects with the general structure of the task.

Subjects performed three sessions consisting of 60 trials in the MRI Scanner. Each trial started with the presentation of the cues lasting for 2000 ms followed by a gap jittered between 2000 and 8000 ms. Subsequently, an outcome was shown for 2000 ms. After the outcome, a rating bar appeared for 4000 ms where subjects had to indicate their beliefs using a MRI-compatible button box. This was followed by an interstimulus-interval (fixation cross) jittered between 1000 and 3000 ms.

### Computational modelling

To derive trial-by-trial regressors for information-theoretic surprise and belief updating, we developed a simple model that performs Bayesian belief updating at each trial. We assume that subjects' prior beliefs at the beginning of a session regard both hidden states to be equally likely:

(1) $$t=1:\;P\left(\theta |m\right)=\left[\begin{array}{cc} \mathrm{vision} & \mathrm{sound} \end{array}\right]=\left[\begin{array}{cc} 0.5 & 0.5 \end{array}\right]\;\text{.}$$

The likelihood of a given outcome depended on the observed auditory and visual cues (*C*_*v*_, *C*_*a*_), which could be good or bad, and a subject's individual belief about the validity of a cue *τ* (free parameter):

(2) $$P\left(o_{t}|\theta ,m\right)=\left[\begin{array}{cc} o_{t}\cdot \tau \cdot C_{v}\; & o_{t}\cdot \left(1-\tau \right)\cdot C_{a}\\ o_{t}\cdot \left(1-\tau \right)\cdot C_{v}\; & o_{t}\cdot \tau \cdot C_{a} \end{array}\right]\;\text{.}$$

The posterior belief about cue contingencies is obtained by an application of Bayes rule at any given trial:

(3) $$P\left(\theta |o_{t},m\right)=\frac{P\left(\theta |m\right)\cdot P\left(o_{t}|\theta ,m\right)}{\sum \left(P\left(\theta |m\right)\cdot P\left(o_{t}|\theta ,m\right)\right)}\;\text{.}$$

The posterior belief at a given trial then serves as a prior belief at the subsequent trial:

(4) $$t\;+\;1:\;P\left(\theta |m\right)=P\left(\theta |o_{t},m\right)\cdot E$$

where *E* encodes subjects' expectations about the frequency of contingency reversal depending on reversal probability *δ*:

(5) $$E=\left[\begin{array}{cc} \delta & 1-\delta \\ 1-\delta & \delta \end{array}\right]\;\text{.}$$

We estimated the two free parameters *τ* and *δ* using (constrained) maximum-likelihood estimation, while constraining *τ* between 0.5 and 1 and *δ* between 0 and 1. To fit our model to behaviour, we used the estimated current belief about the cue contingencies as a predictor for the observed rating and specified the fitting algorithm (using the Matlab-routine fmincon) to minimise the residuals in that linear model (or, equivalently, maximise the explained variance *R*^2^ in observed behaviour).

Based on this model-fitting, we derived trial-by-trial measures of shifts in beliefs formalised as Bayesian surprise, i.e., the Kullback–Leibler divergence from prior to posterior belief $KL\left[P\left(\theta |o_{t},m\right)||P\left(\theta |m\right)\right]=\sum P\left(\theta |o_{t},m\right)\cdot \log\left(\frac{P\left(\theta |o_{t},m\right)}{P\left(\theta |m\right)}\right)$ as well as surprise defined as − *lnP*(*o*|*θ*, *m*).

### Imaging data acquisition and analysis

We acquired *T*_2_*-weighted echo planar images (EPIs) by using a 32-channel head coil at a 3-T Trio Siemens scanner at the Wellcome Trust Centre for Neuroimaging. We acquired a partial volume consisting of 42 3-mm slices in descending order (echo time: 0.065 ms, repetition time: 2.940 ms) at an angle of 30° in the anterior–posterior axis to optimize sensitivity to prefrontal regions (Deichmann et al., 2003). Images were acquired with a field of view of 192 × 192 mm (matrix 64 × 64) resulting in a notional in-plane resolution of 3 × 3 × 3 mm. In each session, at least 245 volumes were acquired. An MR-compatible button box recorded right index and middle finger presses to move the cursor on the rating bar. Auditory cues were presented using MRI-compatible headphones. Foam head-restraint pads were used to minimise head movement and respiratory and cardiac activities were measured and used as covariates in the imaging analysis. Whole-brain 1 mm × 1 mm × 1 mm *T*_1_-weighted structural images were acquired and coregistered with mean EPI images.

We performed preprocessing and all statistical analyses using SPM12b (Wellcome Trust Centre for Neuroimaging, London, UK, http://www.fil.ion.ucl.ac.uk/spm). To allow for *T*_1_ relaxation effects the first 6 functional images were discarded. fMRI time series were realigned to the mean image and unwarped using fieldmaps generated by the Fieldmap toolbox as implemented in SPM12b (Hutton et al., 2002). EPIs and structural scans were normalised and smoothed using a 6 mm^2^ full-width at half-maximum (FWHM) Gaussian kernel using the DARTEL toolbox (Ashburner, 2007), allowing the comparison of data between subjects in common anatomical space.

To investigate for effects in the dopaminergic midbrain, we used a hand drawn region of interest based on previous literature (Schwartenbeck et al., 2014a) for small volume correction of our fMRI analyses.

We conducted a standard mass-univariate analysis to interrogate the data for model-based effects. We created a GLM with separate events for fixation crosses, cue onset, outcome presentation and rating bar onset, modelled as stick functions with duration 0. The outcome regressor was modulated by five additional parametric regressors, namely surprise (z-scored), shifts in beliefs (KL-divergence, z-scored), the difference between estimated shifts in beliefs and the behavioural shift in rating (z-scored), current monetary return and reward prediction error, i.e., a mismatch between expected and observed reward, where the former was defined as the sum of the valence of the auditory and visual cues multiplied by the current belief about cue contingencies as indicated by subjects' rating at the previous trial. The full correlation matrix for surprise, shifts in beliefs, monetary outcome and reward prediction errors is displayed in Table 1. To control for any confounding effects of reward anticipation or confidence on effects at outcome presentation, we included subjects' current belief about the relevant modality (as indicated by the rating at the previous trial), the confidence of these beliefs as indicated by the cursor position at the previous trial, expected (relevant) value (as described above) and expected *irrelevant* value (i.e., the valence of cues multiplied with subjects' beliefs that the cue is *not* relevant, as indicated by the rating at the previous trial) as parametric regressors at cue onset. Furthermore, we included the number of button presses as parametric regressor on the rating regressor. Note that we did not serially orthogonalise these regressors, thus removing all shared variance from the analysis.

We additionally included motion, respiratory and cardiac information as physiological covariates of no interest and included temporal derivatives (first-order differences) to account for slice-timing effects. Furthermore, an AR (1) model was used to account for serial correlations in the fMRI time series and we applied a 128-s cutoff high pass filter.

A standard summary statistic approach was used to test for second-level effects using one-sample t-tests on the estimated responses of the first-level (within-subject) analysis. Random field theory was used to correct for multiple comparisons.

## Results

In a training period prior to the experiment, subjects learned that in both visual and auditory domains one ‘bad’ cue predicted a monetary loss whereas one ‘good’ cue predicted a monetary win, where cues had a validity of 90%. Subjects were instructed that these contingencies remained constant throughout the entire experiment. Thus, for the entire experiment subjects knew the identity of the ‘bad’ shape and tone that predicted a probable loss, as well as the ‘good’ shape and tone that predicted a probable win (stimulus contingencies are depicted in Fig. 1a). Each trial started with a simultaneous presentation of one of the two cues from the visual and auditory domains, followed by an outcome, which was either a monetary win or loss (varying randomly between 10 and 30 pence). Current earnings were indicated by a progress bar at the bottom of the screen.

**Fig. 2:**
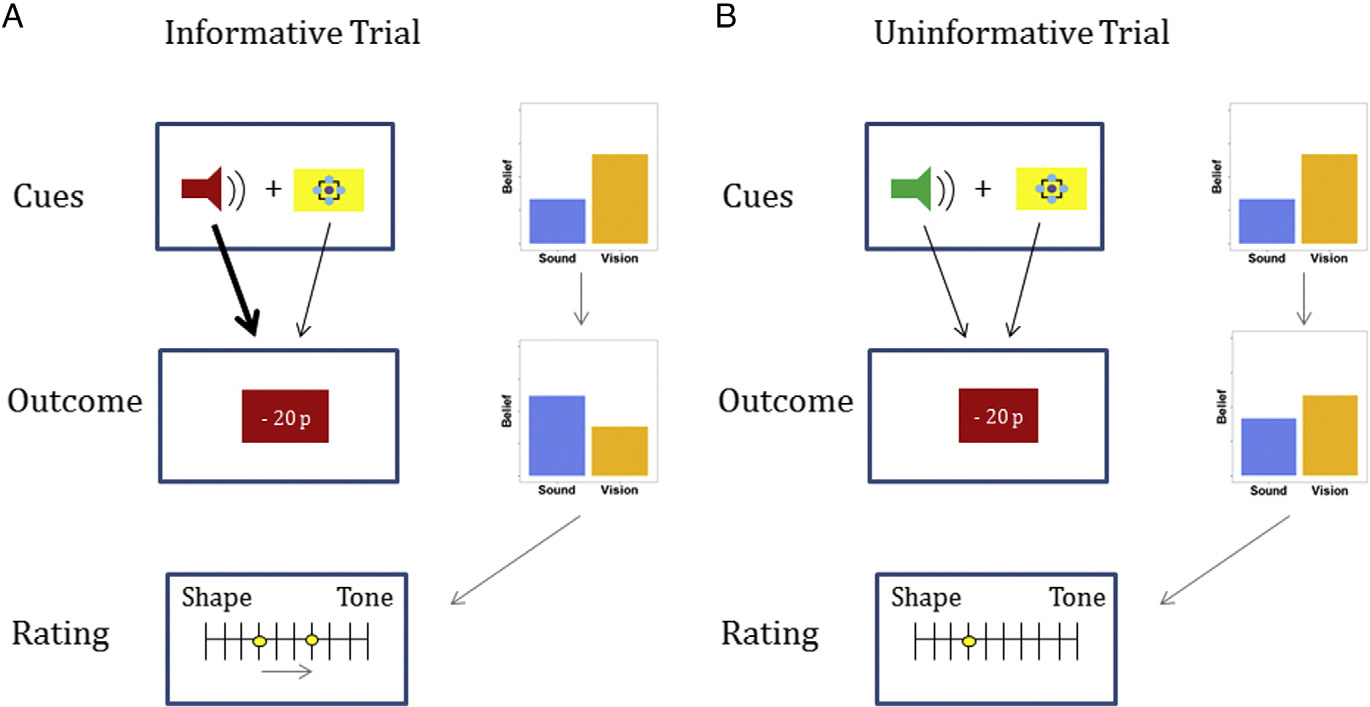
Difference between Bayesian and information-theoretic surprise. (A) Illustration of an informative trial with a shape predicting a win and a tone predicting a loss, assuming that the agent beliefs that visual information is relevant (thus expecting a win as a trial outcome). Observing a monetary loss will contradict the agent's expectations and induce a shift in beliefs about cue contingencies, since the trial outcome was correctly predicted by the tone but not by the shape. This is an illustration of a trial with high Bayesian surprise (i.e., a shift in beliefs). (B) Conversely, two ‘good’ cues followed by a (unexpected) negative trial outcome will not induce a shift in beliefs because the outcome was neither predicted by the tone nor by the shape. Thus, despite the observation of an unexpected outcome the trial provides no meaningful information content and is thus completely uninformative. Note that both examples yield high information-theoretic surprise (i.e., an unexpected outcome is observed), but only in informative trials actual shifts in beliefs can occur. Crucially, each session consisted of the same number of informative and uninformative trials, thus providing a decorrelation between information-theoretic and Bayesian surprise.

The experiment was divided into 60 trial sessions, with reversals occurring between five and six times in a session. Importantly, in half of the trials the cues were uninformative, and both cues were either good or bad (congruent valence of cues). In the other half of the trials cues were informative, i.e. one cue was good and the other bad (incongruent valence of cues), providing a decorrelation of information-theoretic and Bayesian surprise (illustrated in Fig. 2).

**Fig. 3:**
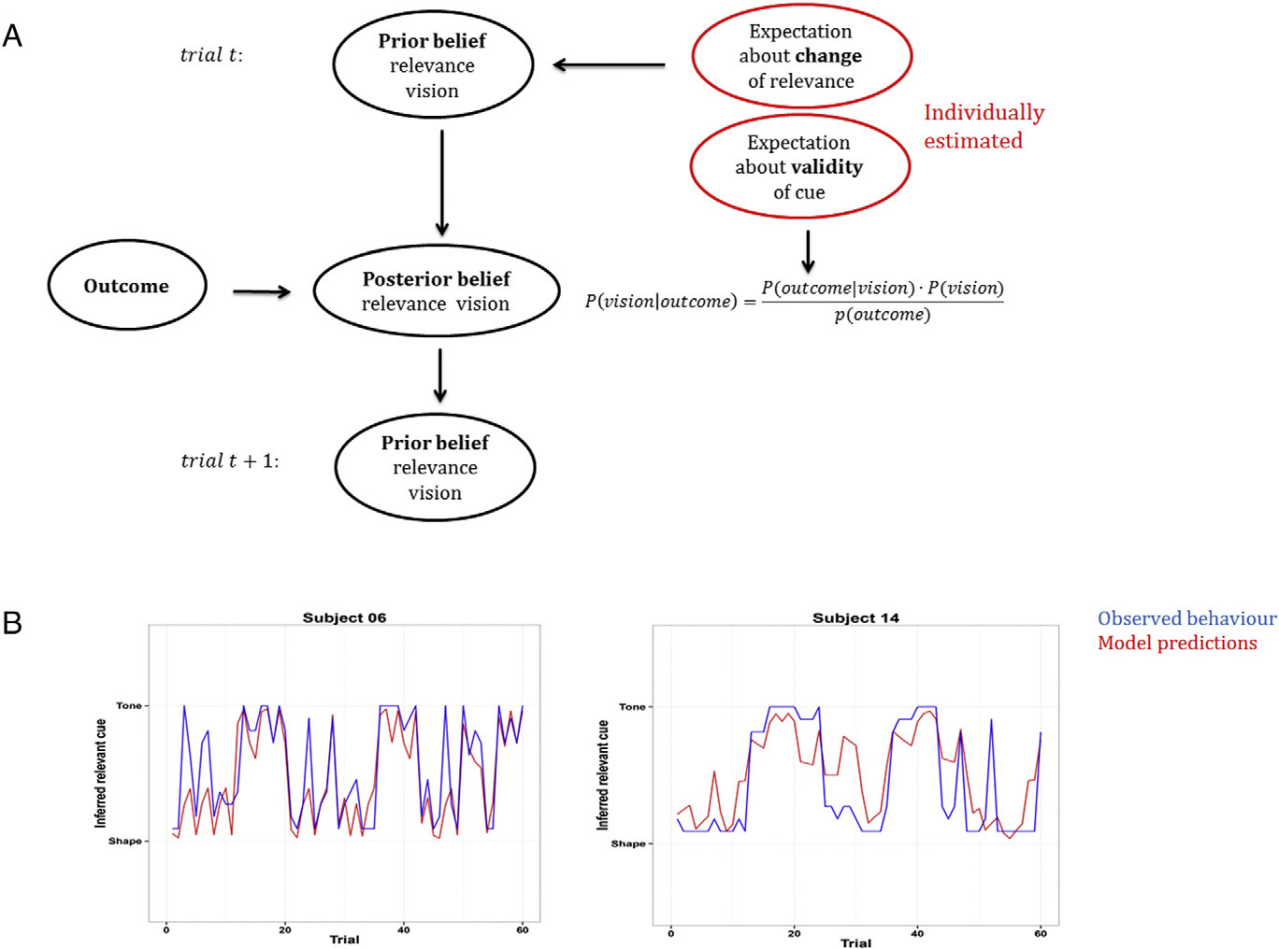
Behavioural modelling. (A) We tested whether subjects updated their beliefs optimally, i.e. updating prior beliefs about cue contingencies to posterior beliefs according to Bayes rule. Note that *P*(*vision*) = 1 − *P*(*sound*). We performed (constrained) maximum-likelihood estimation of subjects' expectation about the frequency of contingency reversal and about the validity of cues. Posterior beliefs at one trial served as prior beliefs at the subsequent trial. (B) We found that our model accounted well for observed behaviour (*R*^2^ = 0.82), indicating that subjects updated their beliefs at least close to optimally (difference of predicted and observed ratings is illustrated in two example subjects).

Subjects underwent an extensive training period to familiarise themselves with the task contingencies, in particular the approximate frequency of reversals (see Methods for details). Subsequently, they performed three sessions of the task during acquisition of fMRI data. To analyse performance on the task, we developed a simple computational model that performs Bayesian belief-updating on a trial-by-trial basis about which modality was currently relevant (model details are depicted in Fig. 3a). To derive trial-by-trial regressors for the use in the imaging analysis we fit individual subjects' data using constrained maximum-likelihood estimation, with subjects' beliefs about the validity of a cue and probability of contingency reversal as free parameters (see Methods for details). We found that our simple model had high accuracy in predicting subject's contingency-ratings (*R*^2^ = 0.82, 95%-confidence interval: [0.97, 0.76]) (see examples in Fig. 3b and Table 2 for a full list of all estimated parameters). Furthermore, we found a high correlation between Bayesian surprise and observed shifts in beliefs (*r* = 0.58, 95%-confidence interval: [0.51, 0.64]). Note that the aim of our computational model was to derive imaging regressors for belief-updates rather than optimally capture behaviour, and as such, we did not compare a range of different models.

To check that subjects were able to adequately perform the task, we correlated subjects' rating at the end of each trial with the true contingencies and found moderate relationship contingencies (*r* = 0.54, 95%-confidence interval: [0.61, 0.45]). This level of performance is in line with the challenging nature of our task. The fact that half of all trials were uninformative, implying that only in half of the trials subjects could actually infer on contingency reversals, naturally induces a delay in tracking changes in current cue-contingencies. To account for this, we additionally compared performance with that of an ideal Bayesian observer (an instantiation of our behavioural model using the true parameters of the task). We found a high correlation between the ideal observer and subjects' performance (*r* = 0.78, 95%-confidence interval: [0.83, 0.71]), confirming that subjects were able to perform the task successfully.

We next derived, based on the model-fitting, trial-by-trial regressors encoding information-theoretic surprise, epistemic value and reward prediction errors, and examined their neuronal correlates. In particular we were interested in whether one of these quantities was encoded in midbrain regions commonly associated with dopaminergic function, namely the Substantia Nigra pars compacta (SNc) and Ventral Tegmental Area (VTA) (Düzel et al., 2009).

**Fig. 4:**
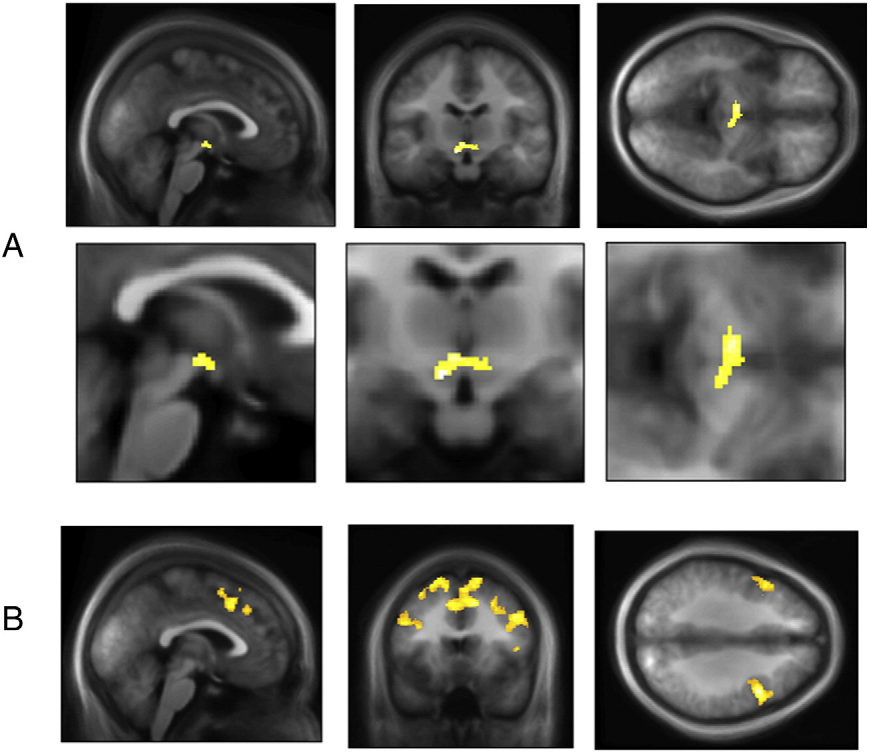
Imaging results. (A) Shifts in beliefs were encoded in dopamine-rich midbrain regions including the Substantia Nigra compacta and Ventral Tegmental Area (small-volume corrected, peak MNI − 6 − 15 − 10, *P*_*Peak*_ < 0.001, *T*_*Peak*_ = 6.24 and peak MNI 10 − 18 − 10, *P*_*Peak*_ = 0.049, *T*_*Peak*_ = 3.64; voxel extend threshold of 100 for display purposes). (B) Information-theoretic surprise was encoded in frontal regions including the dorsal cingulate cortex (peak MNI 12 16 42, *P*_*cluster*_ < 0.001, *T*_*Peak*_ = 4.86; image thresholded at *p* = 0.01 and voxel extend threshold of 392 for display purposes).

In keeping with our hypothesis, we found that dopamine-rich midbrain regions encoded Bayesian surprise or epistemic value (small-volume corrected, peak MNI − 6 − 15 − 10, *P*_*Peak*_ < 0.001, *T*_*Peak*_ = 6.24 and peak MNI 10 − 18 − 10, *P*_*Peak*_ = 0.049, *T*_*Peak*_ = 3.64 see Fig. 4a). To control for additional effects of belief updating beyond estimated Bayesian surprise, we used the observed change in rating at the end of each trial as an overt behavioural proxy for belief updating. We included the difference between estimated Bayesian surprise and the observed change in rating as an additional regressor to test whether midbrain activity predicted shifts in beliefs over and above those predicted by our model. We detected additional effects in the dopaminergic midbrain for belief updating over and beyond our model predictions (small-volume corrected, peak MNI − 10 − 20 − 10, *P*_*Peak*_ = 0.006, *T*_*Peak*_ = 4.65), suggesting that midbrain activity predicted fluctuations in behaviour over and above those predicted by the model. Furthermore, we replicated these effects in the midbrain in an additional analysis, where we used the actual observed shifts in rating as opposed to the belief-updates as estimated by our model (small-volume corrected, peak MNI − 6 − 15 − 10, *P*_*Peak*_ = 0.03, *T*_*Peak*_ = 3.87 and, reaching trend-significance, peak MNI 12 − 18 − 10, *P*_*Peak*_ = 0.065, *T*_*Peak*_ = 3.48). Taken together, both sets of findings provide convergent evidence that putative dopaminergic midbrain regions encode epistemic value, or meaningful information, of a stimulus.

In a whole brain analysis, we found effects for the difference between behavioural rating and modelled shifts in beliefs in the inferior frontal cortex (peak MNI 45 8 21, *P*_*cluster*_ < 0.001, *T*_*Peak*_ = 4.84), the posterior parietal cortex (peak MNI 40 − 72 32, *P*_*cluster*_ = 0.006, *T*_*Peak*_ = 5.05) and in the anterior cingulate cortex (ACC) (peak MNI 6 27 27, *P*_*cluster*_ < 0.001, *T*_*Peak*_ = 5.27). This is in keeping with previous work which also implicates these areas in model updating (O'Reilly et al., 2013), and suggests possible cortical targets of, or inputs to, midbrain neurons encoding epistemic value.

**Fig. 5:**
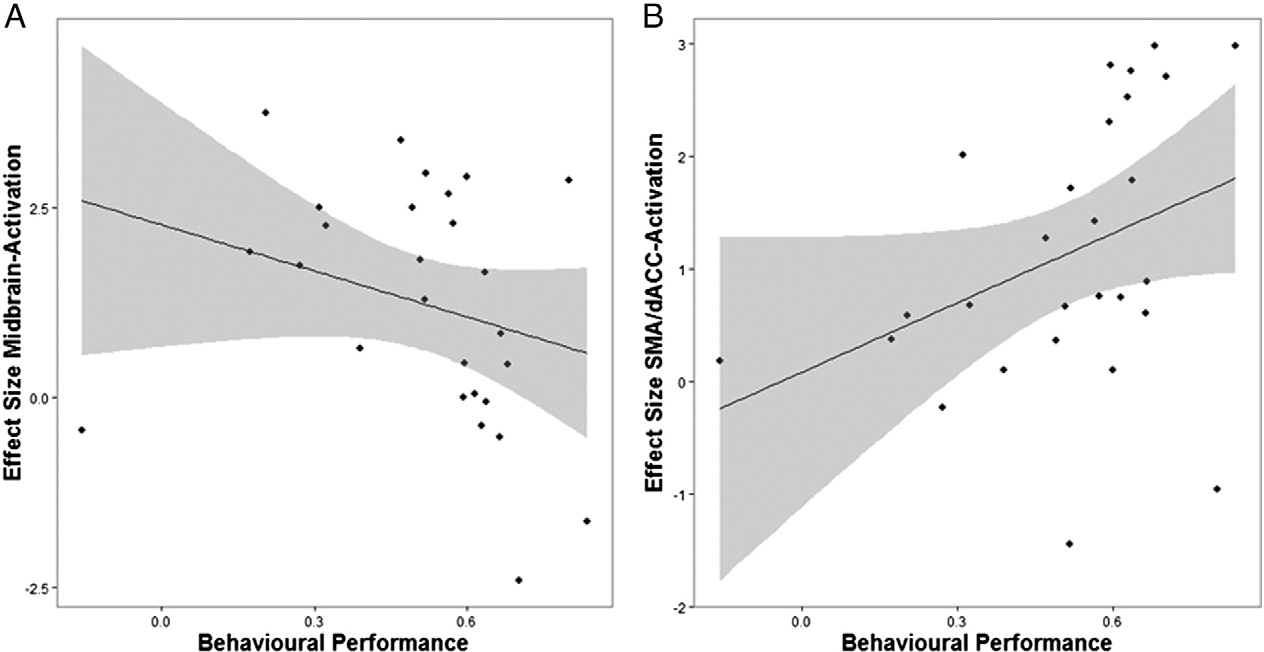
Relationship between performance and midbrain as well as SMA/dACC activation. A) Subjects who were comparatively better (performance on x axis) at inferring the actual cue contingencies displayed relatively less midbrain activation for shifts in beliefs compared to subjects who were worse at tracking these contingencies during the task. B) In contrast, subjects who were comparatively good at tracking the cue contingencies showed higher activity in the SMA/dACC for information-theoretic surprise.

Next, we investigated whether individual differences in performance would relate to the effect size of midbrain activity for meaningful information. As a measure of behavioural performance, we correlated each subject's rating with the true contingencies at any given trial. As a proxy for the effect size of the midbrain activation, we extracted individual betas from the peak-voxel of the combined effects for estimated and observed shifts in beliefs (peak MNI − 9 − 20 − 12, *P*_*Peak*_ = 0.02, *T*_*Peak*_ = 4.10). We found that this measure of performance correlated negatively with the effect size of activation in midbrain regions for shifts in beliefs (Spearman's Rho *ρ* = − 0.44, *p* = 0.02, see Fig. 5a), suggesting that subjects who performed comparatively well in tracking the true contingencies displayed decreased effects in midbrain regions for shifts in beliefs. A similar result (reaching trend-significance) was found when we tested the relationship between performance and the effect size of midbrain activation using a correlation between subjects' behaviour and the ideal observer model as measure of performance (Spearman's Rho *ρ* = − 0.36, *p* = 0.07). This raises the intriguing possibility that individual differences in dopaminergic sensitivity to informative cues might affect learning performance, which might explain the abnormal belief formation that has been associated with dopaminergic abnormalities (Howes and Kapur, 2009).

Consistent with previous findings, effects of information-theoretic surprise were observed in a network of frontal regions including the ventral pre-supplementary motor cortex (pre-SMA) and dorsal anterior cingulate cortex (peak MNI 12 16 42, *P*_*cluster*_ < 0.001, *T*_*Peak*_ = 4.86, see Fig. 4b). This is in keeping with the general idea of the brain as a predictive organ (Dayan et al., 1995, Rao and Ballard, 1999), and supports a distinction between the neuronal processing of meaningful information and simple surprise.

We again tested whether individual differences in the effect size of SMA/dorsal cingulate activation would relate to behavioural performance. Intriguingly, we found a strong positive relationship between behavioural performance and individual effect size of activation for surprise (betas extracted from peak voxel MNI 12 16 42, see Fig. 5b), both for performance with respect to the true task contingencies (Spearman's Rho *ρ* = 0.48, *p* = 0.01) and the ideal observer (Spearman's Rho *ρ* = 0.59, *p* = 0.001). Put simply, this implies that subjects who were comparatively good at tracking the task contingencies showed increased effect sizes of activation in the SMA/dACC for information-theoretic surprise.

Finally, we tested for monetary outcome and reward prediction error effects at outcome time, which are typically associated with activity in the dopaminergic midbrain and its targets such as the ventral striatum (O'Doherty et al., 2004, Schultz, 2012, Schultz et al., 1997). Even at a very liberal threshold of p < 0.05 uncorrected we did not detect effects for those regressors in dopaminergic midbrain or striatal regions (see Table 3 for details). While the absence of significant effects cannot be taken as evidence against the neuronal encoding of reward prediction errors, these results are in line with the general structure of our paradigm wherein subjects were over-trained on the task contingencies. Specifically, subjects had to perform inference on the relevance of cues based on trial-outcomes without a need to perform any kind of reward learning. Our results suggest that, across subjects at least, the dopamine system was if at all only weakly engaged by the reward component of the task, compared with its epistemic aspects, most likely due to the fact that the value per se of outcomes was irrelevant for guiding future behaviour. This finding is in line with evidence that prediction responses to reward in regions such as the striatum are most prominent in situations where they have relevance for on-going behaviour (FitzGerald et al., 2014, Klein-Flügge et al., 2011).

## Discussion

We provide evidence that activity in dopamine-rich midbrain regions encodes the quantity of meaningful information imparted by a stimulus. This is often referred to as its epistemic value (Friston et al., 2015), defined as the Kullback–Leibler divergence between beliefs before and after observing the stimulus. These results support suggestions that dopaminergic rich regions play a key role in belief updating, consistent with the broader notion that abnormal belief formation is associated with hyperdopaminergic function (Kapur, 2003). It also highlights the importance of dopamine for the expression of cognitive flexibility (Bestmann et al., 2014, Van Holstein et al., 2011). We also found evidence for a dissociation between information-theoretic surprise, which is mainly encoded in frontal regions, and actual shifts in beliefs which are associated with activity in putatively dopaminergic midbrain regions. Thus, in keeping with prior work, we show that information-theoretic surprise was encoded in regions typically associated with internal conflict and task difficulty, such as the pre-SMA and dorsal cingulate cortex (Duncan, 2010, Kool and Botvinick, 2013).

In a post-hoc analysis, we detected a between-subject effect, such that participants who were better at tracking the true contingencies during the task displayed smaller effect sizes in terms of shifts in beliefs induced activation within dopamine-rich midbrain regions. Speculatively, this might relate to the effort of tracking the true contingencies or indeed the amount of meaningful information imparted by an observation, such that incoming information is more ‘meaningful’ to subjects who perform poorly on tracking the true contingencies, requiring a stronger update of beliefs compared to subjects whose beliefs in the contingencies are more accurate in the first place. Alternatively, subjects who perform poorly in the task may be hypersensitive to unexpected events and shift their beliefs too often, where this hypersensitivity is reflected in stronger midbrain activation. In either case, our results raise the possibility that inter-individual differences in dopaminergic function drive altered belief formation, over and above any effects of the dopamine system in signalling reward (Schultz et al., 1997). Intriguingly, we found the opposite pattern for effect sizes of activation for information-theoretic surprise in the supplementary motor area and dorsal anterior cingulate cortex: subjects who were comparatively better at tracking the task contingencies showed higher activation for surprising outcomes. While one has to be cautious with post-hoc explanations, this may indeed speak for a between-subjects anticipation effect: subjects who were better at anticipating and tracking reversals of the task contingencies displayed attenuated effects in the midbrain, which may reflect less requirement of an update or, put differently, observations may have become less meaningful with respect to the subject's beliefs. Surprising events, however, cannot be anticipated — and thus may elicit stronger effects in subjects who are better at tracking the true contingencies. Furthermore, this might also indicate that subjects who performed poorly at tracking the true contingencies found it harder to dissociate between surprising and meaningful information, which is a promising topic for future investigations and translational approaches.

Existing work reports a dopaminergic response to novel and unexpected stimuli (Bromberg-Martin et al., 2010, Bunzeck and Düzel, 2006, DeYoung, 2013, Horvitz, 2000). Since these stimuli will, in general, lead to significant belief-updating (Barto et al., 2013) they should also, on an epistemic value account of dopaminergic firing, occasion significant responses. A key contribution of our study is to show, by separating surprise from actual shifts in beliefs, that dopamine is sensitive only to meaningful information, rather than the simple unexpectedness of a stimulus. This highlights an important role for dopamine in belief formation over and above other proposed roles such as reward learning (Schultz, 2012, Schultz et al., 1997), motivational salience (Berridge, 2007), exploration (DeYoung, 2013, Redgrave and Gurney, 2006) and the precision of action selection (Friston et al., 2014a). Further, the dissociation between surprise and meaningful information has also been reported in the context of memory reconsolidation, such that prediction error or surprisal has been shown to control the balance between memory reconsolidation and new memory formation (Sevenster et al., 2014), highlighting the important interplay between memory reconsolidation and dopamine function.

Our findings also resonate with previous work showing that dopamine neurons encode reward uncertainty, displaying highest sensitivity for maximal uncertainty, i.e. a probability of 0.5 for receiving a reward (Fiorillo et al., 2003). Intuitively, it seems plausible that a shift in beliefs relates to higher uncertainty about the environment and thus higher uncertainty about the delivery of a reward. However, as displayed in Fig. 2a, shifts of beliefs can also occur in informative trials when there was relatively little uncertainty about the outcome. Furthermore, beliefs can also shift towards confirming current expectations about the cue contingencies. In tasks where surprise and model updating are highly correlated a surprising outcome will necessarily induce a shift in beliefs as well as higher uncertainty about the task contingencies. The crucial contribution of this study, however, is a dissociation of surprise from belief updates, showing that only the latter elicits midbrain activation whereas the former is associated with effects in the SMA and dorsal anterior cingulate cortex.

Our paradigm also relates to what has been reported as the ‘gambler's fallacy’ or the ‘Perruchet effect’. In brief, this fallacy reflects a failure in acknowledging the independence of events in decision-making paradigms, leading to the overestimation of the likelihood for winning a gamble or receiving a shock as expressed in subjects' beliefs. In contrast, previous work has provided evidence for a second, more automated learning system expressed in the galvanic skin response, which correctly tracks the true contingencies (McAndrew et al., 2012, Weidemann et al., 2009). In our paradigm, however, subjects were asked to track cue contingencies on a trial-by-trial basis where the independence assumption was explicitly violated; the likelihood for a reversal of cue contingencies increased with ongoing time. Therefore, the question of interest becomes how subjects actually represent the ‘non-independence’ of trials in such situations. While this is an interesting question, testing this would be beyond the scope of the present study, which aimed at dissociating belief updates due to meaningful information and the pure unexpectedness of an observation as reflected in information-theoretic surprise. Testing for the representation of those dependencies between trials and its potential physiological correlates, such as skin conductance or pupil diameter, however, remains an interesting area for future research.

In a previous study using a saccadic eye movement task, surprise has been associated with activity in the inferior posterior parietal cortex, whereas model updating was associated with the anterior cingulate cortex (ACC) (O'Reilly et al., 2013). While we partly replicated the findings of an ACC-involvement in model updating, we did not detect any parietal activity for the unexpectedness of an observation. These differences are likely to reflect the lack of a clear motor planning component to our task. Conversely, O'Reilly et al. do not report activity in the midbrain relating to model-updating. While it is dangerous to over-interpret a negative finding, one crucial difference is that in O'Reilly et al. ‘surprise’ and ‘model-update’ trials were explicitly indicated, whereas in our paradigm the epistemic value of feedback cues had to be inferred. The aspect of inferring the epistemic value (meaningful information content) of a stimulus may be the crucial factor determining an involvement of the dopaminergic system. Also the fact that we only detected a weak signal in the ACC is likely to be due to the different nature of tasks, such the ACC typically is more strongly involved in instrumental tasks (Matsumoto et al., 2007, O'Reilly et al., 2013, Rudebeck et al., 2008) whereas our task only required subjects to make inferences based on observations.

Our data provide the first association between information signalling and dopamine putative function in humans. Understanding the link between dopamine and belief updating that implicates a selective sensitivity for meaningful information also sheds new light on findings that associate dopamine with signalling advance information (Bromberg-Martin and Hikosaka, 2009). In the light of our results, dopamine-rich regions might be thought of in terms of encoding advance information not because information is inherently rewarding per se. Instead advance information has epistemic value for an agent in terms of inducing a shift in its beliefs. Our data, therefore, speak to a direct association between dopamine and an agent's beliefs about the world, predicting dopaminergic function will reflect a sensitivity for meaningful information that affects beliefs.

Finally, understanding the link between dopamine and belief formation can provide crucial insight into how dopaminergic abnormalities in conditions such as in schizophrenia, obsessive compulsive disorder and addiction (Friston et al., 2014b, Howes et al., 2009, Howes et al., 2011a, Howes et al., 2011b, Schwartenbeck et al., 2014b, Roiser et al., 2013, Shaner, 1999). In particular, it is widely hypothesised that dysregulated dopaminergic function leads to increased salience being conferred on sensory stimuli, a step in a path leading to psychosis (Howes and Kapur, 2009) (though the precise mechanisms by which this might occur remain to be mapped out). Our results nuance this hypothesis by suggesting that the increased salience conferred by dysregulated dopaminergic activity may be best understood in terms of the pathological assignment of meaningfulness or epistemic value to sensations. This is remarkably consistent with the phenomenology of prodromal psychosis (the so-called ‘Truman sign’) (Fusar-Poli et al., 2008), and provides an intriguing and potentially important topic for future research. By implicating dopamine in signalling the meaningful information imparted by an observation (or, equivalently, the degree of belief updating that is necessary in response to it), our findings suggest new directions for future theoretical and empirical work on the expression of aberrant beliefs.

## Figures and Tables

**Table 1:** Correlation matrix for surprise, shifts in beliefs, monetary outcome and reward prediction errors (RPE).

	Surprise	Shifts in beliefs	Monetary outcome	RPE
Surprise		0.581	− 0.001	0.008
Shifts in beliefs			− 0.006	− 0.003
Monetary outcome				0.589

**Table 2:** Model parameters. Individual beliefs about cue validity and expectations of reversal probability per session estimated individually based on observed behaviour.

Subject	*τ*_*sess* 1_	*τ*_*sess* 2_	*τ*_*sess* 3_	*δ*_*sess* 1_	*δ*_*sess* 2_	*δ*_*sess* 3_
1	0.99	0.99	0.77	0.99	0.77	0.68
2	0.95	0.98	0.92	0.98	0.99	0.94
3	0.69	0.50	0.91	0.72	0.71	0.94
4	0.50	0.7	0.94	0.94	0.99	0.99
5	0.92	0.84	0.86	0.99	0.98	0.98
6	0.98	0.94	0.93	0.83	0.74	0.75
7	0.99	0.79	0.94	0.98	0.83	0.99
8	0.99	0.97	1.00	0.99	0.96	0.93
9	0.83	0.66	0.84	0.96	0.95	0.91
10	0.99	0.99	0.98	0.99	0.99	0.99
11	0.92	0.89	0.77	0.99	0.98	0.88
12	0.82	0.84	0.85	0.97	0.97	0.96
13	1.00	0.99	0.50	0.97	0.72	0.97
14	0.99	0.79	0.95	0.97	0.94	0.96
15	0.83	0.72	0.80	0.91	0.89	0.87
16	0.96	0.86	0.99	0.99	0.97	1.00
17	0.68	0.78	0.91	0.92	0.91	0.94
18	0.88	0.95	0.86	0.96	0.96	0.90
19	0.95	0.96	0.67	0.97	0.97	0.89
20	0.55	0.50	0.97	0.99	0.96	0.99
21	0.97	0.72	0.97	0.96	0.84	0.95
22	0.82	0.89	0.99	0.97	0.98	1.00
23	1.00	1.00	1.00	0.99	1.00	0.99
24	0.92	0.87	0.50	0.99	0.99	0.89
25	0.96	1.00	0.90	0.94	0.88	0.86
26	0.89	0.89	0.84	0.98	0.98	0.95
27	0.74	0.73	0.81	0.97	0.95	0.97

**Table 3:** Effects of reward prediction error on the whole brain level (reported for p < 0.05 uncorrected).

Region	Cluster size	FWE-corrected p-value	T-value peak voxel	Peak coordinates (MNI)		
X	Y	Z
Precentral gyrus	317	0.001	5.22	33	− 12	58
